# Breastfeeding Beyond Six Months: Evidence of Child Health Benefits

**DOI:** 10.3390/nu16223891

**Published:** 2024-11-14

**Authors:** Anita Froń, Magdalena Orczyk-Pawiłowicz

**Affiliations:** Division of Chemistry and Immunochemistry, Department of Biochemistry and Immunochemistry, Wroclaw Medical University, M. Skłodowskiej-Curie 48/50, 50-369 Wroclaw, Poland; magdalena.orczyk-pawilowicz@umw.edu.pl

**Keywords:** prolonged breastfeeding, physiological nutrition, biochemical composition of breast milk, immune properties, mother–breastmilk–infant triad, health outcomes

## Abstract

Breastfeeding is globally recognized as the optimal method of infant nutrition, offering health benefits for both the child and the mother, making it a public health priority. However, the potential advantages of breastfeeding extend well beyond initial months. Breast milk adapts to the evolving needs of the growing infant, and its immunological, microbiological, and biochemical properties have been associated with enhanced protection against infections and chronic diseases, improved growth and development, and lower rates of hospitalization and mortality. This review explores the evidence supporting the continuation of breastfeeding beyond six months. More meticulous studies employing consistent methodologies and addressing confounders are essential. This will enable a more accurate determination of the extent and mechanisms of the positive impact of prolonged breastfeeding and allow for the implementation of effective public health strategies.

## 1. Introduction

The initial 1000 days of life is the most important period in shaping an individual’s health trajectory. During this crucial window, various physiological, immunological, and neurological developments occur, laying the foundation for long-term well-being [1,2,3,4,5,6]. Among the myriad of factors influencing these developmental processes, nutrition stands out as a pivotal element. Breastfeeding, in particular, has been extensively studied and lauded for its profound impact on both immediate and future health outcomes.

Breast milk serves as a critical link between maternal and offspring health, representing a unique biological system known as the ‘connected triad’. This triad, formed by the interactions among the mother, breast milk, and infant, creates a dynamic and interdependent relationship. Each variation within this triad can significantly influence the trajectory of both infant development and maternal health, highlighting the extensive impact of breastfeeding on overall well-being [7,8,9].

Breastfeeding provides a unique blend of nutrients and bioactive components tailored to meet infants’ developmental needs [10]. Children breastfed for extended periods experience lower rates of infectious morbidity and mortality. Growing evidence suggests that longer breastfeeding durations may also protect against overweight, diabetes, allergies, and other chronic illnesses later in life, and contribute to fewer dental malocclusions and higher intelligence scores compared to shorter breastfeeding durations or no breastfeeding at all. These benefits persist into later stages of life [1,11,12,13,14,15]. As previously established, breastfeeding not only benefits infants but also offers considerable advantages for maternal health. It can lower the risk of breast, ovarian, and endometrial cancers, and potentially reduce the risk of diabetes, hypertension, and hyperlipidemia [16,17,18]. The act of breastfeeding not only provides essential nutrition but also fosters emotional closeness through intimate skin-to-skin contact. This physical proximity during breastfeeding has been shown to positively influence infants’ vital signs, indicating a calming effect and enhancing the overall bonding experience between mother and child [19]. Research indicates that mothers who encounter breastfeeding challenges may experience difficulties in forming strong attachments with their infants during this period. Conversely, longer durations of breastfeeding have been found to correlate significantly with higher levels of attachment security [20,21].

In recent years, there has been growing evidence of the substantial health benefits of prolonged breastfeeding, defined as breastfeeding beyond six months [22]. Despite recommendations from organizations like the World Health Organization (WHO), the Association of Women’s Health, Obstetric and Neonatal Nurses, and the US National Academy of Sciences, which advocate exclusive breastfeeding for the first six months and continued breastfeeding for at least one year, actual breastfeeding practices often fall short [19,23,24].

The CDC’s Breastfeeding Report Card for 2022 indicates that among infants born in 2019 in the territory of the United States, a significant majority (83.2%) began breastfeeding, with 78.6% still receiving some breast milk at one month. At six months, 55.8% of infants continued to receive breast milk, while only 24.9% received it exclusively [25]. The data from Europe present a less encouraging picture. A 2021 study, which included data from six European countries (Belgium, Bulgaria, Germany, Greece, Poland, and Spain), revealed that although 85% of children were breastfed at some point, only 6.3% were exclusively breastfed for the first six months. Factors such as lower maternal education, smoking during pregnancy, pre-pregnancy overweight, and younger maternal age were associated with shorter durations of exclusive breastfeeding [26]. If all children were breastfed within an hour of birth, exclusively fed breast milk for the first six months, and continued breastfeeding until the age of two, approximately 800,000 child lives could be saved annually. However, worldwide, less than 40% of infants under six months old are exclusively breastfed [27].

Human milk continues to provide significant nutritional and immunological value beyond 6 months. Studies indicate that the macronutrient content of milk changes to meet the growing child’s energy demands [28,29]. This milk retains high concentrations of immunoglobulins and other bioactive components, highlighting its continued importance for infant health. Promoting extended breastfeeding, even after introducing solid foods, should be a public health priority to prevent infections in infancy [30].

This paper aims to consolidate and present the current evidence on the health benefits of breastfeeding beyond six months.

## 2. Changes in Immune Factors in Breast Milk During Prolonged Lactation

Prolonged lactation is associated with significant changes in the concentrations of various immune factors in breast milk. In the later stages of lactation, especially as complementary foods that provide nutritional value are introduced, breast milk primarily supplies bioactive factors [10,28,31]. These changes are crucial for maintaining the health and development of the infant, offering continued immune protection as breastfeeding extends beyond the initial postpartum months.

### 2.1. Secretory IgA (sIgA)

The concentration of sIgA in breast milk generally remains stable throughout the first 18 months of lactation, averaging around 1.8 g/L [32,33,34]. However, Ongprasert et al. [35] found a positive correlation between the duration of lactation and sIgA concentration. Their study showed that mean total IgA levels were lowest in the first 6 months (1.11 ± 0.14 g/L) and increased in subsequent periods, reaching up to 1.27 ± 0.15 g/L at 18–24 months. According to Goldman et al. [36], both total and secretory IgA concentrations show a slight increase from 0.8 ± 0.3 g/L at 12 months to 1.1 ± 0.3 g/L at 13–15 months, maintaining this level up to 16–24 months (1.1 ± 0.3 g/L for total IgA and 1.1 ± 0.2 g/L for secretory IgA). Additionally, Perrin and colleagues [37] found that sIgA concentrations increase between 11 and 17 months, with a monthly change magnitude of +6.0%. As per the most recent analysis [30], the lowest concentration of sIgA was observed in the first year of lactation, averaging at 2.12 ± 0.62 g/L. After the second year, sIgA levels peaked at 7.55 ± 7.16 g/L. Over prolonged lactation, sIgA levels also positively correlated with IgG concentrations. The ratio of sIgA to protein remained stable for the first two years but significantly increased in the third year [30].

### 2.2. Lysozyme

The concentration of lysozyme in colostrum starts high at 87 mg/L, drops to its lowest point of 24 mg/L at 2–4 weeks postpartum, and then rises progressively over the next five months to reach 245 mg/L [34]. However, from 5 months to 12 months, there is a decrease in lysozyme levels, which are recorded to be 196 ± 41 mg/L at 12 months [34]. Subsequently, lysozyme levels increase to 244 ± 34 mg/L at 13–15 months [36,38]. Nevertheless, there is a slight decrease to 187 ± 33 mg/L by 16–24 month [36,37]. According to the data collected by Perrin et al. [37], between 11 and 17 months, lysozyme shows a monthly change magnitude of +10.2%, indicating a consistent increase during this period. The progressive increase in lysozyme concentration compensates for the decreasing milk volume, resulting in a significant rise in the amount of lysozyme ingested by the infant over time [32].

### 2.3. Lactoferrin

During the first year of lactation, lactoferrin (Lf) concentrations experience a pronounced decrease. Despite this reduction, the infant’s daily intake of Lf remains substantial. By the end of the first year, lactoferrin levels stabilize, continuing to provide significant antimicrobial activity [38]. The mean Lf concentration is lowest during the first 12 months, at 3.39 ± 1.43 g/L, and increases significantly to 5.55 ± 4.00 g/L during the 13–18-month period (*p* < 0.006). Concentrations then remain relatively stable, at approximately 5.02 ± 2.97 g/L for the 19–24-month lactation group and 4.90 ± 3.18 g/L for those lactating beyond 24 months [39]. Lactoferrin concentration in breast milk is positively correlated with protein concentration over the course of lactation (r = 0.3374; *p* = 0.0002) [39]. According to Goldman et al. [36], from 12 to 24 months, Lf concentrations in breast milk increase steadily, starting at 1.0 ± 0.2 g/L at 12 months, rising to 1.1 ± 0.1 g/L between 13 and 15 months, and reaching 1.2 ± 0.1 g/L from 16 to 24 months, while Perrin et al. [37] found that between 11 and 17 months, lactoferrin concentrations rise by 9.7% per month. In light of the analyzed studies, the differences in Lf concentrations in human milk can be attributed to the various measurement methods employed by researchers. Methodological differences, including collection frequency, time of day, and storage conditions, likely contribute to the variations in reported lactoferrin concentrations.

### 2.4. Other Immune Factors

IgG concentrations are lowest during the first year of lactation, averaging at 14.71 ± 6.18 mg/L, and increase after the second year to 18.95 ± 6.76 mg/L. The ratio of IgG to protein decreases progressively from the 1st to the 48th month of lactation [30]. In contrast, the concentration of IgM remains relatively stable during the first two years of lactation, averaging at 2.81 ± 2.74 mg/L. Unlike sIgA and IgG, the ratio of IgM to protein does not show significant changes across different lactation periods [30]. Additionally, the concentrations of other immune proteins, such as C3 and C4, decrease during the first 12 months of lactation [38].

These compositional changes in breast milk ensure that it consistently meets the evolving nutritional and immunological needs of the infant throughout extended lactation. This adaptability indicates that as lactation progresses, the immunoprotective properties of breast milk are not only preserved but may also be enhanced, thereby supporting and promoting the health of the breastfeeding child. No infant formula can fully replicate the complex immunological qualities of breast milk. From an economic perspective, prolonged breastfeeding presents a cost-effective solution, providing ongoing immune protection without the need for expensive supplements or fortified formulas [11,40,41]. This highlights the superiority of extended lactation as a protective measure, ensuring optimal immune support for children in a way that artificial products cannot match.

## 3. Materials and Methods

For this narrative review, we conducted comprehensive English-language literature research for original and review articles published until August 2024 in the PUBMED/Scopus databases. We searched for the following terms, alone or in combination: human milk, breast milk, breastfeeding, breast-feeding, breast feeding, lactation, nutrition, infant, childhood, prolonged, duration, beyond 6 months, 12 months, macronutrients, benefits, advantages, and well-being. We found 486 related articles. The relevant studies were identified by evaluating the abstracts, and complete articles were obtained in cases where abstracts were unavailable. Duplicate papers were removed, and the data were screened to exclude irrelevant works. Case reports, comments, conference papers, commentaries, surveys, and animal studies were all excluded from the full-text publications. Additional manual searches were conducted on the indicated bibliographies, taking into consideration the articles’ novelty, quality, and clinical significance. After applying the exclusion criteria, 179 full-text manuscripts were assessed for eligibility with the consensus of the authors.

## 4. Limitations and Gaps in the Current Research

The current literature on the benefits of prolonged breastfeeding reveals a nuanced and sometimes contradictory picture. While numerous studies suggest various positive effects, the quality and reliability of these findings are often compromised by methodological limitations and insufficient control for confounding variables. A significant issue with existing research is the lack of adequate control for confounding factors such as socioeconomic status, maternal education, and pre-existing health conditions. Furthermore, much of the data available come from studies conducted several years ago, with some articles dating back over 40 years. These older studies may not reflect current breastfeeding practices or advancements in research methodologies. As a result, there is a need for updated and comprehensive studies to validate and extend these earlier findings.

**Figure 1:**
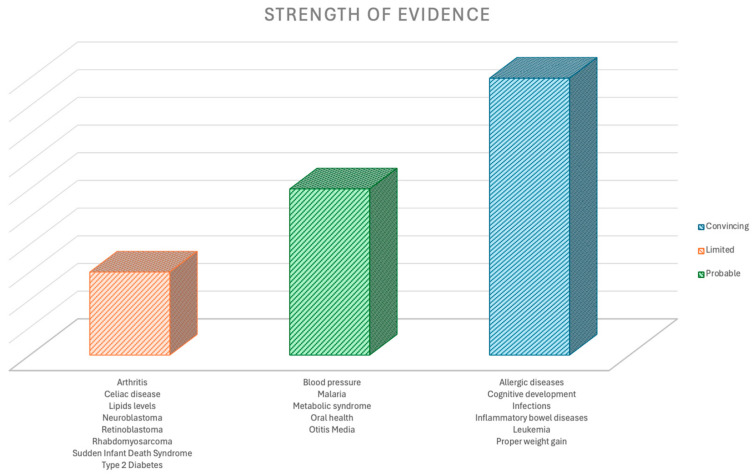
Strength of scientific evidence on health conditions which may benefit from prolonged breastfeeding.

To provide a clearer overview of the current state of evidence, Figure 1 illustrates the strength of scientific evidence on various health conditions that may benefit from prolonged breastfeeding. This illustration serves a conceptual purpose, representing the “strength” as a reflection of the quantity of studies available on each topic.

## 5. Prolonged Breastfeeding’s Effect on Health Outcomes

Research indicates that extended breastfeeding can positively impact a range of conditions, from infectious diseases to chronic illnesses. By examining these conditions, we aim to provide a comprehensive understanding of how prolonged breastfeeding contributes to better health outcomes. This exploration will draw attention to the potential protective effects of breastfeeding throughout infancy and show the importance of continued breastfeeding in the broader context of child well-being and development.

### 5.1. Allergic Diseases

The rising prevalence of allergic diseases in children has become a significant public health challenge globally. This trend is particularly alarming in pediatric populations. Recent data highlight a dramatic increase in allergic conditions, with asthma affecting between 1% and 20% of children, allergic rhinitis ranging from 1% to 18%, and skin allergies between 2% and 10% across various populations [42]. This escalation in allergy rates has been linked to several elements, including improved sanitary conditions, changes in delivery methods, increased antibiotic use, and shifts toward a Western-style diet. These factors not only influence the immune system directly but also impact the gut microbiota, potentially leading to dysbiosis—a microbial imbalance that may play a crucial role in the development of allergic diseases [43,44,45,46].

Bener et al. [47] observed that children breastfed for more than 6 months had a lower risk of allergic diseases than those breastfed for less than 6 months. Specifically, the prevalence rates of asthma, ear infections, wheezing, allergic rhinitis, and eczema were generally lower in children with prolonged breastfeeding. The prevalence of asthma was 15.1% in children with a short breastfeeding duration compared to 14.5% in those breastfed for longer periods. Similarly, rates were lower for ear infections (28.1% vs. 21.8%), wheezing (10.7% vs. 8.4%), allergic rhinitis (21.5% vs. 21.2%), and eczema (18.6% vs. 13.4%). These findings suggest that breastfeeding for more than 6 months may offer protective benefits against various allergic conditions during childhood.

Building on earlier research, Ehlaye et al. [48] found that infants breastfed for more than 6 months had lower prevalence rates of eczema (19.4%), allergic rhinitis (22.6%), and wheezing (12.7%) compared to those breastfed for shorter durations. This suggests that prolonged breastfeeding and exclusive breastfeeding (EBF) reduce the incidence of eczema and allergic diseases in children, even when there is a maternal history of allergy.

Furthermore, Saarinen et al. [49] followed infants with varying breastfeeding durations to assess the incidence of atopic diseases over the first 3 years of life. They found that infants breastfed for more than 6 months had a lower incidence of severe atopic dermatitis and food allergies compared to those breastfed for shorter durations or fed cow’s milk-based formulas early. This protective effect was especially pronounced in children with a family history of atopy. Then, Saarinen et al. [50] conducted a long-term follow-up study of these 236 infants to investigate the impact of breastfeeding on atopic diseases from infancy through adolescence. They found that prolonged breastfeeding (>6 months) was associated with a lowest prevalence of eczema at ages 1 and 3 years compared to shorter durations of breastfeeding. This protective effect extended into adolescence, with the prolonged breastfeeding group showing a lower prevalence of overall atopy and substantial atopy at age 17 compared to the groups with intermediate (1–6 months) or minimal (<1 month) breastfeeding. These studies suggest that breastfeeding for over 6 months may help prevent atopic diseases throughout childhood and adolescence. Additionally, breastfeeding duration has a greater impact on reducing atopy than family history alone, especially in children consuming cow’s milk, highlighting the role of dietary and other factors in promoting breastfeeding’s protective effects.

Complementing previous findings, the study by van Ginkel et al. [51] examined how breastfeeding duration relates to the risk of clinical food allergies. The study included 492 participants, with breastfeeding durations ranging widely from less than 1 month to 42 months. Participants were categorized into quartiles based on breastfeeding duration, showing that longer breastfeeding appeared to provide a protective effect against food allergies. They found that for each additional month of breastfeeding, there was a 4% decrease in the risk of developing clinical food allergies to any type of food. However, it is important to note that they reported no significant association between breastfeeding (versus bottle-feeding) and food allergies, even after correcting for confounding factors.

Obihara et al. [52] discovered that children who were breastfed for longer durations (≥6 months) had a significantly lower risk of allergic diseases overall, particularly hay fever. The study showed a clear inverse relationship between breastfeeding duration and the incidence of allergic diseases, with adjusted odds ratios of 0.50 (95% CI: 0.31–0.82) for allergic disease and 0.53 (95% CI: 0.29–0.99) for hay fever. However, this association was not statistically significant for asthma (adjusted OR: 0.67; 95% CI: 0.31–1.49) or eczema (OR: 0.56; 95% CI: 0.29–1.08). Similar conclusions were drawn by Huang et al., who found that exclusive breastfeeding for more than 6 months was significantly associated with a reduced risk of hay fever (0.93, 95% CI: 0.89–0.97) and eczema (0.96, 95% CI: 0.93–0.99). Overall, the longer the duration of breastfeeding and the more exclusive it was, the lower the prevalence of these diseases [53].

The KOALA study conducted in the Netherlands investigated the relationship between the age of first introduction of cow’s milk and other food products and atopic manifestations in the first two years of life, considering breastfeeding duration as a confounder. They found that delayed introduction of food products was associated with a higher risk of recurrent wheeze. Nevertheless, a longer duration of breastfeeding (7–9 months) was linked to a reduced risk of recurrent wheeze, with a similar trend observed for breastfeeding durations longer than 9 months. The study observed a statistically significant trend, indicating that longer breastfeeding duration is associated with a reduced risk of recurrent wheeze [54].

While exclusive breastfeeding for at least the first six months of life is widely promoted and recommended, the relationship between breastfeeding and the prevention of food allergies presents a more nuanced picture [19,24]. In populations where peanut allergy prevalence is notably high, the European Academy of Allergy and Clinical Immunology (EAACI) Task Force suggests that the optimal age for introducing peanuts is between four and six months as part of complementary feeding. The same timeframe is recommended for introducing eggs into the diet. A critical aspect of this strategy is that the protective effects associated with the early introduction of allergenic foods are most pronounced when breastfeeding continues for at least eight months. This highlights the importance of combining prolonged breastfeeding with the timely introduction of allergenic foods to maximize potential benefits. Moreover, the EAACI guidelines advocate against the avoidance of dietary allergens during pregnancy and breastfeeding as a preventive measure for food allergies. This recommendation underscores the evolving understanding of food allergies and their relationship with infant feeding practices. However, the best way to prevent food allergies remains unknown, necessitating more thorough research with robust diagnostic standards [55].

#### 5.1.1. Asthma

It has been established that longer durations of exclusive and any breastfeeding are significantly associated with reduced risks of adverse respiratory outcomes at 15 months. Adjusted analyses in Silvers et al.’s study [56] showed that each additional month of exclusive breastfeeding decreased the risk of diagnosed asthma by 20%, wheezing by 12%, inhaler use by 14%, ‘wheeze AND diagnosed asthma AND inhaler’ by 24%, and current asthma by 21%. Similar reductions were observed for each month of any breastfeeding, albeit at slightly lower percentages. Subsequent research on the same cohort revealed that the protective effect of each month of exclusive breastfeeding decreased over time. Initially observed to offer 21% protection at 15 months, this effect diminished to approximately 9% by 6 years of age [57]. Comparable conclusions were obtained by von Kobyletzki [58], who discovered that infants breastfed for up to 6 months have a 57% higher risk of developing asthma compared to those breastfed for longer durations. However, the confidence interval indicates that this result may not be statistically significant.

The study conducted by Wickman et al. [59] evaluated 4089 children for diagnosed asthma at 2 years of age. They observed a statistically significant advantage associated with breastfeeding exclusively for ≥6 months compared to <3 months (odds ratio 0.67, 95% confidence interval 0.5–0.91). Likewise, Borba et al. [60] confirmed that breastfeeding—whether partial or exclusive—for more than 6 months was significantly associated with a reduced risk of asthma.

In line with these observations, Watanabe and colleagues [61] found that longer durations of breastfeeding, particularly 10–14 months, 14–19 months, and over 19 months, were associated with reduced odds of asthma in a Japanese cohort compared to breastfeeding for less than 10 months. The adjusted odds ratios for asthma were 0.69 (95% CI: 0.52–0.91), 0.73 (95% CI: 0.56–0.97), and 0.67 (95% CI: 0.51–0.88), respectively, for these breastfeeding durations, indicating a protective effect regardless of exclusivity.

Furthermore, according to Al-Makoshi et al. [62], full breastfeeding is linked to a decreased incidence of childhood wheezing and potentially asthma. Extended periods of full breastfeeding, particularly lasting 6 to 12 months or more, were associated with lower likelihoods of mothers reporting that their child had ever wheezed or experienced wheezing within the past year. Furthermore, children breastfed for over 12 months had a lower prevalence of reported asthma.

#### 5.1.2. Allergic Rhinitis

In a large population study analyzing data from 1374 children participating in the Allergic Rhinitis Cohort Study for Kids (ARCO-kids study), long-term breastfeeding (≥12 months) was significantly associated with a lower prevalence of allergic rhinitis (AR). Specifically, compared to short-term breastfeeding (<6 months), long-term breastfeeding (≥12 months) was linked to a reduced prevalence of AR (aOR, 0.54; 95% CI, 0.34 to 0.88). However, the study found that breastfeeding for 6–11 months did not show a statistically significant relationship with the risk of AR [63].

Other studies also confirm that breastfeeding for more than 6 months significantly reduces the risk of AR [53,64]. Additionally, it was found that breastfeeding for less than 6 months increased the risk of developing asthma by 57%, and asthma, in turn, increased the odds of developing rhinitis nearly threefold [58].

### 5.2. Inflammatory Bowel Diseases

Over the past few years, inflammatory bowel diseases (IBDs), such as Crohn’s disease (CD) and ulcerative colitis (UC), have emerged as global health concerns with rapidly increasing occurrence rates [65]. The incidence of pediatric-onset ulcerative colitis ranges from 1 to 4 per 100,000 per year in most regions of North America and Europe [66]. For Crohn’s disease, the prevalence in the United States is estimated at 58 per 100,000 children [67]. Reports indicate that breastfeeding can reduce the prevalence of CD and UC. This protective effect is attributed to breast milk’s ability to shield infants from gastrointestinal infections, as well as IBD, by promoting the development and growth of the gastrointestinal mucosal system along with enhancing the immune system’s ability to remember and respond to pathogens [68,69,70].

Rigas et al. [71] conducted a study involving 68 patients with CD, 39 patients with UC, and 202 control subjects. Breastfeeding durations were as follows: ≤5 months, 6–11 months, and ≥12 months. Their findings indicated a negative association between breastfeeding and the incidence of both CD and UC, with a duration-dependent trend observed. The results showed that breastfeeding was negatively associated with Crohn’s disease (*p* < 0.04) and ulcerative colitis (*p* < 0.07), with relative risk estimates around 0.5. There was also evidence of duration-dependent trends in both conditions, with the greatest reduction in the risk of Crohn’s disease observed with breastfeeding for at least 12 months. Similar conclusions were drawn by Gearry and colleagues [72] based on research involving 638 CD patients, 653 UC patients, and 600 matched controls. The study found that being breastfed was associated with a reduced risk of developing both CD (adjusted odds ratio [OR] 0.55, 95% confidence interval [CI] 0.41–0.74) and UC (adjusted OR 0.71, 95% CI 0.52–0.96). There was a duration–response effect observed for both diseases, indicating that breastfeeding for more than 3 months was protective. However, the lowest risk of developing both diseases was noted when children were breastfed for more than 12 months. Other articles emphasizing the protective effects of breastfeeding for more than 12 months on IBDs include Ng et al. [73] and Xu et al. [74].

A different research project that supports these findings is paper by Ko et al. [75], which reported a reduced risk of CD with breastfeeding for ≥3 months and a decreased risk of UC with breastfeeding for ≥6 months. The study showed a clear duration–response effect, indicating that longer periods of breastfeeding were strongly associated with lower risks of developing IBDs. Another study indicating that a longer breastfeeding duration might be associated with a reduced risk of developing CD is the study by Bergstrand et al. [76]. Additionally, articles by Decker et al. [77], Hansen et al. [78], and Hlavaty et al. [79] suggest a similar protective effect for both IBDs. Lee et al. [80], on the other hand, highlights the protective effect of breastfeeding for ≥6 months specifically for UC.

### 5.3. Otitis Media

Shaaban et al. [81] found that children with acute otitis media (AOM) had been breastfed for a significantly shorter period (8.6 months) compared to healthy controls (13.7 months). Notably, 25% of children with ear infections had been breastfed for less than 6 months, while only 10% of the control group had a similar breastfeeding duration, resulting in an odds ratio of 3. This indicates that children with acute otitis media were three times more likely to have been breastfed for less than 6 months. The study suggests that shorter breastfeeding durations are a major risk factor for developing acute otitis media in early childhood.

Moreover, breastfeeding for up to 11 months significantly prevents AOM, with the strongest protective effects seen within the first 4 months, dropping in the 5th month, and then rising again from the 6th to 8th month. The protective effect decreases but remains statistically significant up to 11 months and positive, though not statistically significant, until 18 months. Furthermore, in their research, Vogazianos et al. [82] concluded that to achieve optimal prevention of AOM, breastfeeding should continue for at least the first 11 months, with some additional benefit extending up to 18 months. The varying importance of breastfeeding corresponds to the child’s developmental stages and changing immunological needs.

In addition to this, Ardc et al. [83] discovered that infants breastfed for longer than 12 months had a lower incidence of acute otitis media compared to those breastfed for less than 12 months (*p* < 0.05). Additionally, exclusive breastfeeding during the first 6 months significantly reduced the occurrence of this condition. Similarly, Weiss [84] noted that breastfeeding for 6 months or longer was most effective in reducing the risk of otitis media (OM), although no continued benefits were observed beyond 2 years of age.

### 5.4. Metabolic Health

The global prevalence of excessive weight gain is an escalating public health concern. Alarmingly, in 2022, over 390 million children and adolescents aged 5–19 years were overweight, including 160 million of them being obese [85]. This rising trend is particularly troubling due to its strong association with a heightened susceptibility to various chronic health conditions, such as cardiovascular disease (CVD), type 2 diabetes (T2DM), and metabolic syndrome (MetS) [86].

MetS, in particular, poses significant health risks, as it consists of a cluster of inter-related metabolic abnormalities. Individuals with MetS typically present with at least three of the following conditions: abdominal obesity, hyperglycemia, hypertension, hypertriglyceridemia, and low levels of HDL cholesterol (HDL-C). The presence of these risk factors, especially when obesity is concentrated in the abdominal area and originates in childhood, shows the critical need for early intervention and preventive measures to combat the growing incidence of MetS and its associated health complications [86,87,88].

#### 5.4.1. Weight Gain

Liu et al. [89] conducted a comprehensive study to evaluate the relationship between breastfeeding duration and body mass index (BMI) in children and adolescents aged 6 to 16 years. The study found a significant negative correlation between breastfeeding duration and BMI after adjusting for various covariates. Specifically, children who were breastfed for more than 12 months had significantly lower BMIs compared to those breastfed for less than 12 months (β = −0.274; 95% CI: −0.422, −0.127; *p* < 0.01). This inverse relationship was consistent across different age groups and genders, though it was particularly pronounced in boys. The study further highlighted that children and adolescents breastfed for more than 12 months had a significantly lower prevalence of obesity compared to those breastfed for shorter durations. Specifically, the adjusted odds ratio (OR) for obesity among participants breastfed for more than 12 months was 0.853 (95% CI: 0.748, 0.974, *p* < 0.05).

Similar findings were reached by Gewa et al. [90], who found that longer breastfeeding durations, particularly beyond 18 months, are significantly associated with lower risks of childhood overweight/obesity (*p* < 0.05). Children breastfed for more than 24 months had a 45% decrease in the odds of being overweight compared to those breastfed for less than 12 months. Nascimento Simon et al. [91] also found comparable results. Their hierarchical multiple analysis revealed that longer breastfeeding durations provide greater protection against overweight and obesity. Specifically, exclusive breastfeeding for six months or more was associated with a significantly lower risk of overweight and obesity, and breastfeeding for more than 24 months further reduced the risk (OR = 0.13; 95% CI [0.05, 0.37]; *p* = 0.00). What is more, similar conclusions were drawn by Fallahzadeh et al. [92], Liese et al. [93], and Von Kries et al. [94].

Grummer-Strawn and colleagues [95] came up with interesting conclusions. They found that longer breastfeeding is not associated with a decrease in mean BMI but rather with a decrease in the standard deviation of BMI, leading to simultaneously lower rates of underweight and overweight. Breastfeeding seems to protect against overweight not by uniformly reducing BMI in all children but by reducing the variability in BMI. However, this study was conducted in 2004, which is relatively dated, and there is a lack of more recent data to corroborate these findings.

#### 5.4.2. Blood Pressure

The study by Hosaka et al. [96] examined how breastfeeding duration influences blood pressure in 7-year-old Japanese children. It categorized mother–offspring pairs into short-term (mean 5.1 months) and long-term (mean 11.3 months) breastfeeding groups, measuring both self-measured home blood pressure (HBP) and conventional blood pressure (CBP). Children in the long-term breastfeeding group exhibited significantly lower HBP compared to those in the short-term group, whereas CBP did not differ significantly between the groups. Breastfeeding for more than 8 months, regardless of birth weight, was strongly linked with lower HBP, underscoring breastfeeding’s protective role against elevated blood pressure in young children. Similar conclusions were drawn by Lin and colleagues [97], who found that the duration of breastfeeding had an inverse relationship with blood pressure values in children entering the first grade. For each additional month of breastfeeding, systolic blood pressure decreased by 0.07 mmHg and diastolic blood pressure decreased by 0.05 mmHg. These differences were statistically significant. Furthermore, breastfeeding for more than 12 months was associated with a reduced risk of hypertension, with an adjusted risk ratio of 0.83 (95% CI: 0.70 to 0.98, *p*  =  0.03).

An interesting relationship was discovered in the study by Liang et al. [98]. Children who were breastfed for 4 to 10 months exhibited the lowest prevalence of hypertension. Conversely, those breastfed for more than 10 months showed an increased risk of hypertension, particularly among rural children. This elevated risk in the rural cohort may be partly due to the fact that 33.86% of these children were breastfed for more than 10 months, which could help explain the higher prevalence of hypertension observed in these areas.

#### 5.4.3. Metabolic Syndrome

Breastfeeding has a universal protective effect. The study by Wang et al. [99] compared two sample populations of young people in Spain and China, highlighting significant differences in metabolic health markers. Spanish children generally exhibited higher mean values for height, weight, BMI, waist circumference, triglycerides (TGs), and systolic blood pressure (SBP) compared to their Chinese counterparts. This disparity was reflected in an elevated prevalence of metabolic risk factors, leading to a higher overall prevalence of MetS (2.5%) compared to Chinese adolescents (0.5%). Breastfeeding duration appeared to be an important factor influencing these outcomes. The Spanish cohort, which was breastfed for a longer duration (9 months on average), showed a significantly more pronounced protective effect of breastfeeding against metabolic risk factors. This included a reduced likelihood of low HDL-C, hyperglycemia, and hypertriglyceridemia, and ultimately, a lower risk of MetS. In contrast, the correlation between breastfeeding duration and these metabolic markers was weaker in the Chinese cohort, suggesting that the benefits of breastfeeding may be significantly modulated by cultural or environmental factors.

Also, Gonzales-Jimenez et al. [100] found that longer breastfeeding durations, especially beyond 6 months, were associated with a reduced likelihood of MetS diagnosis in both males and females. Additional risk factors for MetS included maternal smoking during pregnancy, along with maternal overweight and obesity. The same conclusions were reached by Esfarjani et al. [101] and Wang et al. [102].

### 5.5. Oral Health

The relationship between prolonged breastfeeding and oral health, specifically its impact on childhood dental caries (ECC) and severe early childhood caries (S-ECC), has been the subject of several studies with varying findings. These studies collectively suggest that while breastfeeding offers numerous health benefits, extended breastfeeding durations may be associated with an increased risk of dental caries, particularly in specific contexts.

Chaffee et al. [103] investigated the association between breastfeeding for 24 months or longer and the prevalence of S-ECC. They found that breastfeeding for 24 months or beyond, especially when done frequently, was associated with the highest adjusted prevalence of S-ECC (0.45; 95% CI, 0.36 to 0.54). In contrast, breastfeeding durations of less than 6 months, 6–11 months, and 12–23 months had lower prevalence rates of S-ECC (0.22, 95% CI: 0.15 to 0.28; 0.38, 95% CI: 0.25 to 0.53; and 0.39, 95% CI: 0.20 to 0.56, respectively). However, the prevalence ratio for ECC with breastfeeding for ≥24 months was 1.17 (95% CI, 0.85 to 1.78), which did not reach statistical significance.

Similarly, Tanaka et al. [104] found a relationship between breastfeeding duration and dental caries prevalence. Compared to breastfeeding for less than 6 months, breastfeeding for 18 months or longer was significantly associated with a higher prevalence of dental caries (adjusted prevalence ratio of 1.66; 95% CI, 1.33–2.06). In contrast, breastfeeding for 6 to 17 months did not show a significant association with caries prevalence, indicating that the risk associated with breastfeeding may increase after 18 months.

Conversely, Nirunsittirat et al. [105] reported that full breastfeeding for 6–11 months was significantly associated with a lower risk of dental caries, evidenced by a lower decayed, missing, and filled surface (DMFS) score (adjusted RR 0.77; 95% CI, 0.63–0.93) and lower caries prevalence (adjusted RR 0.45; 95% CI, 0.22–0.90).

Adding to the body of evidence, Peres et al. [106] observed that children breastfed for 24 months or more had a higher prevalence of dental caries and a 2.4 times greater risk of S-ECC compared to those breastfed for up to 12 months. The risk of dental caries among children who were breastfed for 13 to 23 months was not significantly different from those breastfed for up to 12 months. Their study underscored the increased risk associated with prolonged breastfeeding and suggested that preventive dental interventions should be introduced early to mitigate caries risk.

Multiple meta-analyses and reviews [107,108,109,110,111] support these findings, indicating that breastfeeding for up to 12 months is associated with a reduced risk of caries. However, breastfeeding beyond 12 months has been reported to be linked to an increased risk of caries. It is essential to consider this relationship in the context of eating habits, feeding hours, and hygiene practices. Even with these considerations, the advantages of breastfeeding beyond six months outweigh the potential issues and harm associated with caries.

### 5.6. Infections

Given the numerous benefits of breast milk, including its potent anti-infective and immunological properties, exclusive breastfeeding has been shown to significantly reduce the risk of infectious diseases during infancy. Diarrheal diseases and acute respiratory infections remain the leading causes of morbidity and mortality among children under five years old globally [112,113,114]. While the protective effects of breastfeeding against infections are well established, it is important to note that there are specific, albeit rare, instances where breast milk feeding should be withheld. These include maternal infections such as HIV, HTLV, viral hemorrhagic fevers, and untreated brucellosis, where the risk of transmission outweighs the benefits of breastfeeding [114].

Extended breastfeeding has the potential to offer a protective effect against hospitalizations during infancy. In their study, Størdal et al. [115] underscore the beneficial impact of breastfeeding beyond 12 months of age. The research revealed that infants breastfed for shorter durations, specifically 6 months or less, had a higher risk of hospitalization (10.0%) compared to those breastfed for 12 months or more (7.6%). After adjusting for various factors, the adjusted relative risk (RR) was 1.22 (95% confidence interval: 1.14–1.31), indicating a significant difference in hospitalization rates. Interestingly, infants breastfed for 6 to 11 months showed similar risks of hospitalization compared to those breastfed for 12 months or longer.

#### 5.6.1. Respiratory Tract Infections

One of the initial findings in this area was reported by Nafstad et al. [116], showing that children breastfed for more than 6 months had a lower incidence of lower respiratory tract infections (LRTIs) compared to those breastfed for less than 6 months and demonstrated that for each month of shorter breastfeeding duration, the odds of LRTIs increased by an average of 5%. The disparity in infection rates between these breastfeeding groups widened with higher levels of maternal smoking. Similar results were obtained by Tromp and colleagues [117], who found that breastfeeding for 6 months or more significantly reduced the risk of lower respiratory tract infections (LRTIs) up to the age of 4 years, with an adjusted odds ratio (aOR) of 0.71 (95% confidence interval [CI]: 0.51–0.98).

It is also worth noting that Li R. et al. [118] found significant health benefits associated with longer breastfeeding durations and higher breast milk intensity (proportion of milk feedings). Children breastfed for 9 months or longer had lower odds of throat (aOR: 0.68) and sinus infections (aOR: 0.47) compared to those breastfed for less than 3 months. Moreover, the likelihood of these infections was reduced by 34% to 50% compared to children who were breastfed for up to 6 months but received formula supplementation before reaching 6 months. Additionally, high breastfeeding intensity (>66.6%) during the first 6 months was linked to lower odds of sinus infections (aOR: 0.53) compared to low breastfeeding intensity (<33.3%).

In addition, Fisk et al. [119] conducted a birth cohort study in the UK and found that longer breastfeeding durations were associated with a reduced risk of respiratory infections in a dose-dependent manner. Breastfeeding for more than 6 months significantly decreased the risk of wheezing, lower respiratory infections, and general respiratory morbidity compared to never breastfeeding (adjusted RR 0.43; 95% CI 0.30–0.61). Each additional month of breastfeeding further reduced the risk of these conditions, with adjusted RRs of 0.88 (95% CI 0.83–0.92) for the first 6 months (*p* < 0.001) and 0.97 (95% CI 0.95–0.99) for 6–12 months (*p* = 0.002).

#### 5.6.2. Gastrointestinal Infections and Diarrhea

Ardc et al. [83] discovered that the incidence of acute gastroenteritis was significantly lower in infants who were breastfed for longer durations. Specifically, infants who were breastfed for more than 12 months experienced fewer instances of acute gastroenteritis compared to those breastfed for less than 12 months (*p* < 0.05). Additionally, infants who were exclusively breastfed during the first 6 months also had a significantly reduced risk of acute gastroenteritis. This suggests that both exclusive breastfeeding in the early months and prolonged breastfeeding can provide protective benefits against gastroenteritis in infants. In line with these observations, Fisk et al. [119] noted that breastfeeding for more than 6 months significantly reduces the risk of gastrointestinal infections, with each additional month of breastfeeding further lowering this risk.

A different approach was taken by Rebhan et al. [120], who examined how breastfeeding duration influences gastrointestinal infections among infants, categorizing them into three groups based on breastfeeding practices: Group A (≥6 months of exclusive breastfeeding), Group B (≥4–6 months of breastfeeding with varying exclusivity), and Group C (no or <4 months of any breastfeeding). Their analysis found that infants exclusively breastfed for 6 months or more had a significantly lower risk of experiencing ≥1 episode of gastrointestinal infections during months 1–9 of life compared to those with shorter durations of breastfeeding (adjusted OR: 0.60; 95% CI: 0.44–0.82). The incidence rates of these infections were 20.4% in Group A, 28.0% in Group B, and 27.9% in Group C. These findings highlight the protective effect of exclusive breastfeeding for at least 6 months against early-life gastrointestinal infections.

To explore the benefits of prolonged breastfeeding, Mølbak et al. [121] assessed its impact on the risk and duration of diarrhea in 849 infants under three years of age. With a median weaning age of 22 months and 25% of children still breastfed at 27 months, the study found that children who had been weaned experienced significantly more diarrheal episodes compared to those who continued breastfeeding past one year. Specifically, weaned children had relative risks of 1.41 and 1.67 for diarrhea at one and two years of age, respectively. Additionally, the duration of diarrheal episodes was one day longer in weaned children compared to those still breastfed at 1 and 2 years of age. The incidence of diarrhea was higher in weaned children than in partially breastfed children, both in one-year-olds (relative risk 1.41; 95% CI 1.23 to 1.62) and in two-year-olds (1.67; 95% CI 1.29 to 2.15). The mean duration of a diarrheal episode was 5.3 days in breastfed children compared to 6.3 days in weaned children (*p* = 0.001). These findings suggest that the beneficial effects of breastfeeding are not restricted to infancy.

After adjusting for various maternal and infant factors, including smoking during pregnancy and age at introduction of solid foods, Fisk et al. [119] discovered that each additional month of breastfeeding reduced the risk of diarrhea. Specifically, the adjusted relative risks (RRs) were 0.88 (95% CI 0.83–0.92) for 0–6 months (*p* for trend < 0.001) and 0.97 (95% CI 0.95–0.99) for 6–12 months (*p* for trend = 0.002). The protective effect of breastfeeding was most pronounced in the first 6 months after birth; however, the study also showed significant benefits associated with extended breastfeeding into later infancy.

Moreover, Ruuska et al. [122] found that breastfeeding for over 6 months reduced the incidence of diarrhea during the first year of life in both atopic and nonatopic infants. However, they observed no significant effect on the overall incidence of diarrhea over the two-year follow-up period, as infants breastfed for longer durations experienced more diarrhea in the second year of life. Additionally, prolonged breastfeeding was associated with reduced severity of diarrhea, specifically among infants aged 7–12 months.

### 5.7. Cognitive Development

One of the first significant insights into this issue was provided by Daniels et al. [123], who conducted a study with 1984 participants and found strong associations between prolonged breastfeeding and improved intellectual growth. The research used a nonverbal intelligence test assessing fluid abilities at 8.5 and 11.5 years of age. At 8.5 years of age, among healthy and low-birthweight children, breastfeeding for 12–18 months was associated with increases in nonverbal intelligence test scores by 1.6 and 9.8 points, respectively. For low-birthweight children, breastfeeding for 18–24 months and ≥2 years led to a 6.6-point and 7.1-point increase in test scores, respectively. After 2 years, the beneficial association with prolonged breastfeeding persisted, albeit weaker. Improved cognitive development was primarily observed in healthy-birthweight children breastfed for 12 to 18 months.

In the study of Duazo and colleagues [124], mothers who breastfed their children for longer periods tended to have lower educational attainment and come from lower-income households. Despite these socioeconomic disparities, breastfeeding duration emerged as a significant predictor of future psychosocial development in late childhood, particularly after adjusting for socioeconomic and related factors. Compared to children breastfed for 5 months or less, those breastfed for longer durations showed higher psychosocial scores. Specifically, among 5-year-olds, children breastfed for 12 months or more scored 2 to 3 points higher on psychosocial assessments compared to those breastfed for less than 6 months. Interestingly, the study noted that the apparent protective effect of breastfeeding peaked during the second year of life and then gradually declined.

While breastfeeding duration did not significantly impact intelligence scores across the entire sample, Slykerman et al. [125] observed notable associations among children classified as small for gestational age (SGA). However, a trend suggested that longer breastfeeding periods correlated with higher intelligence scores overall. Among SGA children, breastfeeding for longer than 12 months was significantly associated with higher IQ scores at 3.5 years. Specifically, children breastfed for more than 12 months had adjusted scores that were 6.0 points higher than those who were not breastfed (*p* = 0.06). Exclusive breastfeeding for 5 months or longer within the SGA group was also significantly linked to higher intelligence scores, showing a 5.9-point increase compared to non-breastfed children. Additionally, the duration of predominant breastfeeding impacts language development. Whitehouse et al. [126] found that children predominantly breastfed for 4–6 months or more than 6 months had significantly higher language scores compared to those breastfed for 0–4 months or not breastfed at all. The study also revealed significant associations between the duration of predominant breastfeeding and language ability at 10 years. Specifically, children breastfed predominantly for 6 months showed a language score increase (β = 4.04).

Furthermore, Jedrychowski et al. [127] found that the duration of exclusive breastfeeding had a positive impact on children’s intelligence quotients (IQs). Children exclusively breastfed for up to 3 months had an average IQ score that was 2.1 points higher compared to others (95% CI, 0.24–3.9). Those breastfed for 4–6 months had IQs higher by 2.6 points (95% CI, 0.87–4.27). Children breastfed for more than 6 months experienced an even greater benefit, with IQs increasing by 3.8 points (95% CI, 2.11–5.45). However, Sajjad [128] suggests that the association between breastfeeding duration and child IQ may largely stem from sociodemographic factors, parental lifestyle, and maternal IQ. In his study, initially, each additional month of breastfeeding conferred an advantage of 0.32 points (95% CI, 0.20–0.44). Yet, after adjusting for child-specific factors and various environmental influences, this association weakened considerably to 0.09 points (95% CI, −0.03 to 0.21).

### 5.8. Leukemia

The study by Shu and colleagues [129] suggests that primarily breastfed children have a reduced risk of childhood acute leukemia, including both acute myeloid leukemia (AML) and acute lymphoblastic leukemia (ALL). A stronger inverse association was observed for children breastfed for more than 6 months (AML: OR = 0.57, 95% CI: 0.39–0.84; ALL: OR = 0.72, 95% CI: 0.60–0.87). Leukemia risk decreased with longer breastfeeding durations, particularly up to 12 months for ALL and 9 months for AML. Although the risk was lower for children breastfed beyond 12 months, this reduction was not statistically significant.

The relationship between the duration of breastfeeding and the risk of childhood leukemia appears to follow a non-linear dose–response pattern. Gong et al. [130] discovered that as the duration of breastfeeding increased, the odds of childhood cancer, including leukemia, significantly decreased. The study found that breastfeeding for a duration of 7–9 months was particularly protective against childhood leukemia, with a significant reduction in risk compared to other breastfeeding periods (OR: 0.498; 95% CI: 0.318–0.780; *p* = 0.002). This suggests that the protective effect of breastfeeding may peak at a specific duration, beyond which the benefits may plateau. This correlation is further supported by the results obtained by Gao et al. [131]. Interestingly, they found that a lower education level among mothers was associated with an increased risk of leukemia in children.

The findings from MacArthur et al. [132] suggest that breastfeeding, particularly for more than six months, provides significant protective benefits against childhood leukemia (OR = 0.78, 95% CI 0.71–0.85). However, introducing a significant amount of milk supplementation (more than 50% of the child’s diet) after six months of age was associated with an increased risk of childhood leukemia, particularly when compared to exclusive breastfeeding. This indicates that while breastfeeding itself is protective, the benefits may diminish or be counteracted when a high proportion of milk supplements are introduced.

There are also other studies that confirm the beneficial impact of breastfeeding for more than 6 months, compared to not breastfeeding or breastfeeding for less than 6 months, in reducing the risk of leukemia. Among these are studies by Cheng et al. [133], Bener et al. [134], Rudant et al. [135], Altinkaynak et al. [136], and Bener et al. [137].

### 5.9. Malaria

Pincelli et al. [138] found that children who received breast milk for ≥12 months, irrespective of complementary feeding practices, were significantly less likely to develop antibodies to blood-stage *Plasmodium vivax*, indicating a lower risk of infection. Breastfeeding for this duration reduced the risk of *P. vivax* seropositivity by 79.8% during the first 2 years of life among children born to mothers who had experienced *P. vivax* infection during pregnancy. Also, Safeukui-Noubissi et al. [139] discovered that prolonged breastfeeding for at least 2 years was associated with a decreased risk of severe malaria in children (OR: 0.57; 95% CI [0.33–0.94]). An intriguing study by Vora et al. [140] revealed that breastfeeding was associated with a markedly lower incidence of malaria (1.36 vs. 2.44, *p* = 0.008) in HIV-exposed children aged 6–15 months who were still being breastfed and receiving TS prophylaxis.

### 5.10. Individual Reports

There are several conditions for which prolonged breastfeeding has shown beneficial effects. However, it is important to note that the existing literature lacks a robust body of reliable evidence, and many of the findings are based on relatively old studies that have not been consistently confirmed by more recent research. Therefore, while there are hints in the literature connecting prolonged breastfeeding with the prophylactic potential of certain conditions, these associations require further investigation. Continued research is essential to determine whether the protective effects of prolonged breastfeeding hold true across different populations and updated methodologies. Additionally, the existing data remain fragmentary and require further research to fully understand the extent of these protective effects.

#### 5.10.1. Arthritis

Research indicates that shorter breastfeeding durations, particularly those of less than 4 months or ending by 6 months, are associated with an increased risk of developing juvenile idiopathic arthritis (JIA). In contrast, exclusive breastfeeding for at least 4 months, followed by continued partial breastfeeding while introducing other proteins, may help reduce the risk of JIA in childhood [141].

#### 5.10.2. Celiac Disease

Breastfeeding during the introduction of dietary gluten has been associated with a reduced risk of celiac disease in children under 2 years old. Specifically, breastfeeding while introducing gluten reduced the risk (adjusted OR: 0.59), and continuing breastfeeding after gluten introduction provided an even greater protective effect (adjusted OR: 0.36). It is noteworthy that this study was conducted in a population where most infants were breastfed for at least six months [142].

#### 5.10.3. Hand, Foot, and Mouth Disease

A study by Li et al. [143] investigating the relationship between breastfeeding duration and the risk of severe hand, foot, and mouth disease (HFMD) revealed that breastfeeding for more than 6 months was significantly associated with a reduced risk of severe HFMD. Specifically, durations of 6–12 months (OR 0.701, 95% CI 0.539–0.913) and over 12 months (OR 0.504, 95% CI 0.341–0.746) were independently linked to a lower risk of severe HFMD after adjusting for confounding factors.

#### 5.10.4. Lipid Levels

Recent research has shown the association between breastfeeding duration and lipid profiles among 12,110 children and adolescents aged 5–19 years across China. They discovered that participants breastfed for more than 12 months exhibited significantly lower levels of total cholesterol (TC), LDL cholesterol (LDL-C), HDL cholesterol (HDL-C), and TC/HDL-C ratios compared to those who were not breastfed. Additionally, children breastfed for more than 12 months also experienced a 43% reduced risk of high TC. This protective effect was particularly notable in children and young adolescents aged 5–14 years, suggesting that a longer breastfeeding duration is linked to lower lipid levels and a decreased risk of abnormal lipids in this age group [144].

#### 5.10.5. Neuroblastoma

Children with neuroblastoma were found to be less likely to have been breastfed compared to control children, with an OR of 0.6 (95% CI = 0.5–0.9). The potential protective effect of breastfeeding against neuroblastoma appeared to strengthen with longer breastfeeding durations, with children breastfed for 13 months or more showing the lowest likelihood (OR = 0.5, 95% CI = 0.3–0.9) [145].

#### 5.10.6. Retinoblastoma

According to the research conducted by Heck and colleagues [146], breastfeeding is associated with a reduced risk of unilateral retinoblastoma, particularly when it lasts for 7–11 months. However, extending breastfeeding beyond 12 months does not appear to offer additional protective benefits, as no dose–response relationship was observed with longer breastfeeding durations.

#### 5.10.7. Rhabdomyosarcoma

The findings by Lupo et al. [147] indicate that breastfeeding for 12 or more months is strongly associated with a reduced risk of childhood RMS (Rhabdomyosarcoma), as demonstrated by the odds ratio (OR = 0.36, 95% CI: 0.18–0.70). Additionally, the significant trend (*p* = 0.01) suggests that as the duration of breastfeeding increases, the risk of childhood RMS decreases further, highlighting a dose–response relationship between breastfeeding duration and reduced RMS risk.

#### 5.10.8. Sudden Infant Death Syndrome

The study by Thompson et al. [148] suggests that a longer breastfeeding duration provides substantial protection against sudden infant death syndrome (SIDS). Both any breastfeeding and exclusive breastfeeding durations exceeding 2 months offer increased protection, with the most significant benefit observed for durations greater than 6 months, which is associated with a 64% reduction in the risk of SIDS.

#### 5.10.9. Type 2 Diabetes

Kue Young et al. [149] explored prenatal and early-infancy risk factors for type 2 diabetes among native Canadians. Their findings indicate that being breastfed for more than 6 months (odds ratio = 0.36; 95% CI: 0.13–0.99) is significantly associated with a reduced risk of developing type 2 diabetes compared to being breastfed for less than 6 months.

## 6. Discussion

**Figure 2:**
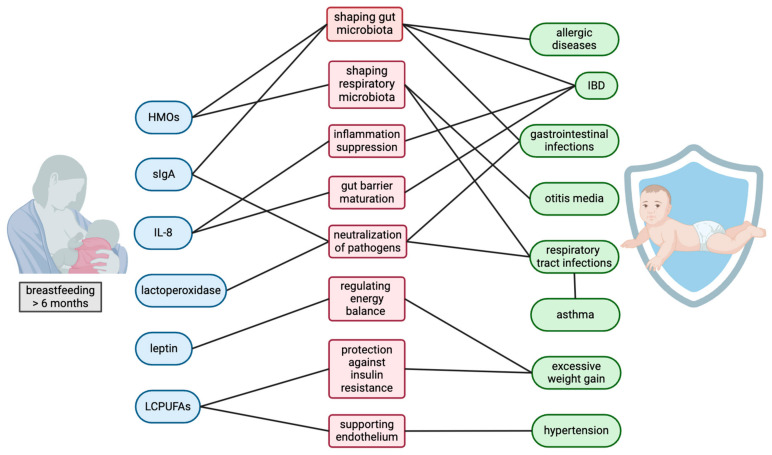
The mother–breastmilk–infant triad: how bioactive components in breast milk contribute to disease protection for infants. Legend: HMOs, human milk oligosaccharides; sIgA, secretory immunoglobulin A; IL-8, interleukin 8; LCPUFAs, long-chain polyunsaturated fatty acids; IBD, inflammatory bowel disease.

Breastfeeding is widely recognized for its numerous health benefits, particularly in protecting infants against infections and impacting the proper growth and development of children. The mechanisms through which breastfeeding imparts these benefits are multifaceted, involving immunological, microbiological, and biochemical processes, and they are depicted in Figure 2.

One of the primary protective mechanisms of breastfeeding is the transfer of sIgA from mother to infant through breast milk. sIgA is resistant to digestion, allowing it to accumulate in the infant’s intestine where it binds to antigens on pathogens, rendering them less infective and protecting against infections [69,150]. This immunological defense is especially crucial during the early months of life when the infant’s immune system is still developing [151]. Interestingly, the absence of passive sIgA in the gut is associated with the upregulation of Gram-negative *Pasteurellaceae* and Gram-positive *Lachnospiraceae*. These bacterial families are often found in higher abundance in the gut microbiota of pediatric patients with inflammatory bowel disease, suggesting a protective role of sIgA against such conditions [152]. Moreover, sIgA antibodies provide protection against gastrointestinal infections caused by microbes like Giardia, ETEC, and *Campylobacter* [153]. Additionally, non-breastfed children exhibit an increased abundance of *Clostridium difficile*, a pathogen linked to allergic sensitization and immune-mediated diseases, protecting against asthma, eczema, wheezing, etc. [68,154,155].

Breast milk plays a significant role in shaping the infant microbiota, promoting the colonization of beneficial bacteria and suppressing the growth of pathogenic bacteria [156,157]. The prebiotic effect of glycans and human milk oligosaccharides (HMOs) in breast milk supports the growth of beneficial bacteria while inhibiting the colonization of pathogens [69,158,159,160,161]. This modulation of the gut microbiota is essential for developing immunologic tolerance and preventing inappropriate immune responses [68,69,161,162,163]. Breastfeeding also shapes the composition of the respiratory microbiota in the nasopharynx [164,165,166].

For instance, the presence of *Lactobacilli* in breast milk contributes to distinct nasopharyngeal colonization, reducing the incidence of otitis media during breastfeeding. This effect is facilitated by the direct bacteriostatic properties of breast milk and the microbiota associated with it, which collectively help in reducing the pathogens responsible for OM [167]. Additionally, breast milk also forms a protective layer on the nasopharyngeal mucosa, shielding against the transmission of bacteria and viruses that cause respiratory illnesses [168]. This may also enhance the protective effect of breastfeeding against asthma development, as reduced respiratory tract infections—well-known risk factors for asthma—are associated with breastfeeding. Furthermore, breastfeeding has been demonstrated to support lung development and improve lung function. Children who were breastfed show increased lung volumes, with this benefit attributed to the mechanical stimulation of suckling at the breast during early infancy, which can prevent asthma [157].

Breast milk contains various bioactive components that contribute to its protective effects. Lactoperoxidase, an enzyme present in milk, catalyzes the formation of hypothiocyanate from saliva thiocyanate, which can kill the bacteria responsible for infections such as OM. This enzymatic activity provides a biochemical defense against pathogenic bacteria, complementing immunological and microbiological protections [167]. Breastfeeding also reduces the overall disease burden, which in turn allows for more resources to be allocated toward combating infections [169]. Moreover, prolonged breastfeeding has been shown to reduce the likelihood of hospital stays in early childhood [115].

Furthermore, breast milk factors can suppress the induction of inflammatory responses, such as IL-8 expression in cultured intestinal epithelial cells, thereby attenuating early inappropriate inflammatory reactions [69]. This anti-inflammatory property of breast milk is crucial in preventing conditions like IBD by promoting the maturation of the gut barrier and supporting the development of immunological memory to pathogens [68,69]. Another reason breastfeeding helps prevent chronic inflammation is that it promotes mother–child attachment, which positively affects cortisol regulation in breastfed infants [157].

Moreover, through its stimulation and modulation of the infant’s immune system, breast milk leads to enhanced vaccine responses and increased thymus size [129]. Additionally, it is worth highlighting that breast milk contains soluble tumor necrosis factor (TNF)-related apoptosis-inducing ligand (TRAIL), which regulates apoptosis and cell proliferation in various tissues, as well as human alpha-lactalbumin made lethal to tumor cells (HAMLET), a substance with known anticancer properties [170]. Together, these components may influence leukemogenesis and reduce the risk of childhood leukemia [129,170].

Infant formulas typically contain higher levels of fats, proteins, and sodium compared to breast milk. This composition can lead to elevated levels of Insulin-like Growth Factor 1 (IGF-1) in the bloodstream. IGF-1 stimulates the proliferation of adipocytes, leading to increased fat accumulation and, consequently, higher risks of obesity. Moreover, breast milk contains bioactive compounds such as leptin, which is crucial in regulating energy balance and appetite. Breast milk’s balance of proteins, fats, and carbohydrates promotes appropriate growth and satiety, preventing overfeeding. Unlike formula, which can lead to higher caloric intake, breast milk’s tailored composition reduces the risk of excessive weight gain [89,171,172]. Additionally, breast milk helps maintain proper blood pressure levels by reducing sodium intake during infancy, providing long-chain unsaturated fatty acids (LCPUFAs) that support the health of tissue membranes and the coronary endothelial system, and protecting against hyperinsulinemia and insulin resistance throughout early life [171,173,174].

Moreover, breastfed infants often have a different trajectory of brain development compared to those who are never breastfed or those with shorter breastfeeding durations. Breastfeeding has been linked to increased gray matter, hippocampal volume, brain activation, and cortical thickness [175,176,177]. It is also important to highlight that HMOs act as prebiotics, supporting the developing gut microbiome, which can help reduce inflammation and promote the production of metabolites affecting brain function through the gut–brain axis. Additionally, HMOs may supply sialic acid, a crucial nutrient for brain tissue organization [178]. These combined effects contribute to enhanced cognitive and neurodevelopmental outcomes in children [175,176,177,178].

The multifaceted benefits of prolonged breastfeeding extend beyond basic nutrition, offering profound and long-lasting effects on an infant’s health and development. The immunological, microbiological, and biochemical properties of breast milk work synergistically to protect against infections, support optimal growth, and enhance proper development.

However, the data regarding the beneficial impacts of prolonged breastfeeding present a complex picture, with many studies failing to adequately control for confounding variables. This underscores the necessity for more rigorous and comprehensive research efforts. There is a significant need for well-designed, prospective studies that employ mixed-method approaches to capture both quantitative and qualitative data. Additionally, cross-cultural research is essential to understand how different societal, environmental, and genetic factors may influence the outcomes of prolonged breastfeeding. Such studies should aim to control for potential confounders such as socioeconomic status, maternal education, and pre-existing health conditions to provide clearer insights. By addressing these gaps, future research can more accurately determine the extent and mechanisms of the protective effects of prolonged breastfeeding on various health outcomes in children.

## 7. Conclusions

Breastfeeding, especially when extended beyond the early months, provides substantial health benefits that significantly influence infant development and well-being. The immunological, microbiological, and biochemical properties of breast milk work synergistically to offer strong protection against infections, reducing rates of hospitalizations and even mortality, promote optimal growth, and support cognitive development and brain function. As breast milk adapts to the evolving needs of the growing infant, prolonged breastfeeding remains a cost-effective means of delivering comprehensive immune and developmental support, surpassing the advantages of costly formulas.

Key findings from the literature highlight that longer durations of breastfeeding are associated with numerous health benefits. These include a reduced risk of gastrointestinal and respiratory infections, better growth and cognitive development, and a lower likelihood of developing allergic diseases and obesity later in life. Additionally, breastfeeding positively impacts metabolic syndrome, blood pressure regulation, otitis media, and malaria. Although less common, there are also isolated reports suggesting the potential advantages of extended breastfeeding for conditions like arthritis, blastomas, celiac disease, and even sudden infant death syndrome. Therefore, it is essential to encourage mothers to breastfeed, particularly to continue breastfeeding for extended periods, as this practice offers significant health benefits for both the infant and the mother and addresses the growing need for effective preventive health measures amid declining breastfeeding rates and rising health issues.

More rigorous and controlled studies are needed to fully understand the complexities and mechanisms of extended breastfeeding. Future research should address potential confounders and include diverse populations to better assess its impact on long-term health outcomes, refine public health recommendations, and improve child health practices.

## Data Availability

The data can be shared upon request.
